# Epidemiology and costs of surgical treatment of developmental dysplasia of hip in the Brazilian Public Health System in a decade

**DOI:** 10.31744/einstein_journal/2021GS5625

**Published:** 2021-11-24

**Authors:** Bruno Gonçalves Schröder e Souza, Bruno Marinho Coelho Vasconcelos, Higor Pereira Pujoni, Mário Círio Nogueira, Valdeci Manoel de Oliveira, Alfredo Chaoubah

**Affiliations:** 1 Universidade Federal de Juiz de Fora Juiz de Fora MG Brazil Programa de Pós-Graduação em Saúde Coletiva, Universidade Federal de Juiz de Fora, Juiz de Fora, MG, Brazil.; 2 Núcleo de Pesquisa em Ortopedia e Traumatologia Hospital e Maternidade Therezinha de Jesus Juiz de Fora MG Brazil Núcleo de Pesquisa em Ortopedia e Traumatologia, Hospital e Maternidade Therezinha de Jesus, Juiz de Fora, MG, Brazil.

**Keywords:** Developmental dysplasia of the hip, Prevalence, Unified Health System, Spatial analysis, Time series studies, Costs and cost analysis, Brazil

## Abstract

**Objective:**

To describe and analyze the epidemiology and costs of surgical treatment of hip dysplasia in the Brazilian Public Health System.

**Methods:**

An ecological analytical study that evaluated a time series and the geographic distribution of surgical treatment of hip dysplasia in Brazil. Frequencies of cases, number of cases and associated factors were analyzed. Correlations, frequency maps and flow maps are presented and discussed.

**Results:**

During the study, 14,584 patients with dysplasia were admitted to hospitals according to Information Technology Department of the Public Health System. Patients underwent hospital treatment specific for dysplasia in 8,592 cases (at an average cost of R$ 2.225,50, total cost of R$ 19.124.086,25– updated values). In this group, mortality rate was 0.046% and mean hospitalization time was 4.41 days (standard deviation of 2,39 days). Age between 1 and 4 years (37.7%), female sex (64.5%) and white race (46%) were more frequent. Greater rates of specialists (R²=0.82; p<0.001), greater proportion of counties with high/very high human development index (R²=0.79; p<0.001), and higher *per capita* income (R²=0.68; p<0.001) correlated to greater rates of treatments undertaken per 1,000 live births (as per State of treatment). The factor most related to treatment rate per 1,000 live births (as per State of residence) was white race (R²=0.90; p<0.001). Southern states had higher treatment rates (as per State of residence, rate of 0.73/1,000), and Southeast states had greater absolute frequency of cases (46.7%) and greater flow of patients.

**Conclusion:**

The surgical treatment of hip dysplasia in Brazil occurs frequently, at relevant costs, and is distributed in a heterogenous and unequal fashion in the Public Health System. Southern states have a higher incidence of cases, and there is an association with racial and socioeconomic factors. There was no large variation in the incidence of cases over time.

## INTRODUCTION

Developmental dysplasia of the hip (DDH) is a condition in which the femoral head has an abnormal relation with the acetabulum, and its severity ranges from cases of instability at birth to dislocation.^(1)^ In the absence of diagnosis or late diagnosis (after the first months of life), treatment becomes more complex, morbidity increases, and the chances of normal development of the hip decrease.^(2,3)^ Therefore, it is an established risk factor for early hip osteoarthritis (before 50 years of age), and a condition of major economic and social impact.^(4)^In a recent study carried out in a teaching hospital in Brazil, a significant knowledge gap was detected among pediatricians and pediatric residents, who are responsible for clinical triage, and the consequent early referral of patients for treatment.^(5)^ In other countries, strategies such as systematic ultrasonography have been implemented to prevent these cases from not being identified at the ideal time.^(6)^ However, the cost-effectiveness of this strategy has been questioned, and this type of screening only seems to be appropriate in places where the prevalence of surgery is high.^(7,8)^ In Brazil, this prevalence does not seem to have been studied yet. In fact, epidemiological data on this disease vary by geographic region.^(9)^ The incidence of DDH is estimated to be 1.5 to 20 per 1,000 live births, and is four to eight times more prevalent in women.^(3,5)^ There is significant variability in incidence among racial groups in the same geographic location. The incidence of clinical neonatal hip instability at birth ranges, for example, from 0.4 per one thousand in Africans, to 61.7 per thousand in Polish Caucasians.^(9)^In Brazil, Puech was the pioneer in discussing the epidemiology of DDH. He argued that in our country, at the beginning of the 20^th^ century, there was a low frequency of this disease, which changed due to migratory currents, mainly from Europe.^(10,11)^ However, no studies have been identified in the last decade exploring the epidemiology of DDH and its treatment. The null hypothesis of this study is the incidence of surgical treatment of DDH in the Public Health System (SUS – *Sistema Único de Saúde*) has a frequency similar to that of other countries, does not vary over the years, and is distributed non-uniformly across the states.

## OBJECTIVE

To describe and analyze the epidemiology and costs of surgical treatment of hip dysplasia in the Public Health System.

## METHODS

This is an ecological analytical study that evaluated the time series and spatial distribution of cases of surgical treatment of DDH in SUS during the period of a decade (between 2008 and 2017), with secondary data collection, based on the analysis of data from the Information Technology Department of the Brazilian Public Health System (DATASUS - *Departamento de Informática do Sistema Único de Saúde*).^(12)^

According to Resolution 510 of 2016 from the National Research Ethics Committee (CONEP - *Comissão Nacional de Ética em Pesquisa*), research using secondary data from public databases with unrestricted access is exempt from obtaining an opinion from the Research Ethics Committee (CEP - *Comissão de Ética em Pesquisa*). This research was developed as part of the Graduate Program in Collective Health at the *Universidade Federal de Juiz de Fora* (UFJF) and the *Núcleo de Pesquisa em Ortopedia e Traumatologia of Hospital e Maternidade Therezinha de Jesus*, in Juiz de Fora (MG, Brazil).

The data files were obtained from the DATASUS platform, including the entire database of the Hospital Information System of SUS (SIHSUS System),^(12)^ the population base (with data from the 2015 Brazilian population estimate),^(13)^ surveys and research National Household Sample Survey (PNAD - *Pesquisa Nacional por Amostra de Domicílios*),^(14)^ and the database of the Live Births Information System (SINASC - *Sistema de Informações sobre Nascidos Vivos*).^(15)^ The data were tabulated and analyzed in the TabWin 4.1.5 software and presented as graphs, tables, and maps. In parallel, we used the 2015 Brazilian medical demography survey,^(16)^ and the registers of specialists of the Brazilian Hip Society (SBQ - *Sociedade Brasileira de Quadril*) and the Brazilian Society of Pediatric Orthopedics (SBOP - *Sociedade Brasileira de Ortopedia Pediátrica*), which contain quantitative data with the number of specialists in these surgeries, by geographical unit.^(17,18)^

The study included all the data from the Hospital Admission Authorizations (AIH - *Autorização de Internação Hospitalar*) performed at SUS, from January 2008 to December 2017.

Among the AIHs included in the study, cases in which there was late treatment of DDH were selected. For this, different selections were made in the parameters of the TabWin software, generating Groups A, B and C.

Group A included all inpatients whose main diagnosis field contained the code Q65 of the International Classification of Diseases and Related Health Problems (ICD-10), which refers to developmental dysplasia of the hip. This parameter aimed to promote a broad search, with good sensitivity for conditions related to diagnosis of dysplasia, although it allowed the inclusion of several cases not necessarily related to late inpatient (surgical) treatment of DDH.

In Group B, an additional filter was included to restrict the cases obtained in the selection of Group A, improving the specificity of the search. The filter consisted of 11 surgical treatments typical of late treatment of DDH with the following SUS codes: 0408040327, 0408060190, 0408040181, 0408040343, 0415010012, 0408040157, 0408040173, 0408040220, 0415020034, 0408040165, and 0415020069, according to the Table of Procedures, Medicines, Orthoses, Prostheses, and Special Materials of the SUS.

In Group C, to remove cases not related to hip sparing surgeries, all cases of patients aged 40 years or older were excluded.

The cases included in Group C made up the final research sample, which was used for all comparisons and analyses. The outcome variables were the annual absolute frequencies and rates of treatments performed per 1,000 live births during the period. Data were tabulated and presented by state and geographic region of treatment and patient residence. The following variables were also evaluated to explore associations: patient age, sex, race, demographic variables (live birth rate, resident population, and *per capita* household income), as well as health care variables (ratio of hospitals with inpatient care per 100,000 inhabitants). Additionally, also included were ratio of medical schools per 100,000 inhabitants, ratio of SBQ and SBOP specialists per 1 million inhabitants, total hospital costs, and costs per admission.

The data were presented as descriptive statistics by year (incidence rates, percentages, means, and standard deviations - SD). To study associations, the rate of surgical treatments performed per one thousand live births, per state, was calculated and bivariate correlation tables were used, using Pearson’s correlation coefficient, with significance level set at 0.05. Statistical tests were performed in the (SPSS) program, version 21.The geographical distribution of treatments is presented by means of maps generated from the data plotted in TabWin software (version 4.1.5).

## RESULTS

From January 2008 to December 2017, a total of 14,584 hospitalizations of patients with a primary diagnosis of hip dysplasia (ICD-10 Q65) were recorded in Brazil at SUS (Group A). We found 68 different types of treatments performed among these patients, many of which were unrelated to the object of this study (for example, cesarean delivery, conservative treatment of fractures, and surgical treatment of polytrauma patients, among others). Table 1 shows the frequency of all treatments performed in Group A and the frequency found in Group B, after applying the treatment filter.For Group B, we used ICD and treatment code as filters, obtaining an absolute frequency of 9,470 cases treated in a decade. In this group, 22 deaths were recorded (mortality of 0.23%), and the mean hospital stay was 5.07 days (minimum of 1.5 and maximum of 9.95 days).Group C, which included an additional age filter, representing the sample of interest in this study (i.e., dysplasia patients undergoing hip sparing surgery), accounted for 8,592 hospitalizations. The nominal amount paid by SUS in a decade was R$ 12.889.988,36. According to the Broad Consumer Price Index (IPCA - *Índice de Preços ao Consumidor Amplo*) corrections for January 2020, the value wasR$ 19.124.086,25 (mean of R$ 2.225,50 per hospitalization). In this group, four deaths occurred (mortality of 0.046%), with a mean hospital stay of 4.41 days (SD=2.39; minimum of 1.5 and maximum of 10.48 days).

**Table 1:** Cases per type of treatment in Group A

Treatment given identified by code and name	Group A n(%)	Included in Group B
0408040327 – Surgical treatment of congenital hip dislocation	2,464 (16.90)	Yes
0408060190 – Osteotomy of long bones except hand and foot	1,149 (7.88)	Yes
0408040181 – Closed reduction of congenital hip dislocation	1,102 (7.56)	Yes
0408040343 – Surgical treatment of spontaneous/progressive/paralytic hip dislocation	996 (6.83)	Yes
0415010012 – Treatment with multiple surgeries	867 (5.94)	Yes
0408040157 – Pelvic osteotomy	845 (5.79)	Yes
0408040173 – Closed reduction with manipulation of spontaneous/progressive dislocation of the hip with application of an orthopedic device	721 (4.94)	Yes
0408040220 – Surgical revision of congenital hip dislocation	475 (3.26)	Yes
0415020034 – Other treatments with sequential surgeries	472 (3.24)	Yes
0408040165 – Osteoplastic reconstruction of the hip	255 (1.75)	Yes
0415020069 – Sequential treatments in orthopedics	124 (0.85)	Yes
57 other types of treatments not related to the scope of this study	5,114 (35.06)	No
Total	14,584 (100)	

Source: prepared by the author based on the data from the Information Technology Department of the Brazilian Public Health System.

Brasil. Ministério da Saúde. Departamento de Informática do Sistema Único de Saúde (DATASUS). Transformação digital para o SUS. Brasília (DF): DATASUS; ©2008 [citado 2020 Abr 24]. Disponível em: http://www2.datasus.gov.br/DATASUS/index.php?area=0901&item=1&acao=25^(12)^

Table 2 shows the time progression of treatment frequencies and costs in Group C. A mean of 947 cases were performed per year (SD=85.80). The trend line for Group C shows an increase by less than 10% in the number of treatments over the decade. In addition, Table 2 also shows other frequency and cost data in Group C. Females were the most frequent (64.5% in Group C). The mean age at hospitalization was 8.14 years (SD=7.63 years). The distribution of costs by age group followed a distribution similar to that of frequency (R^2^=0.99). The over-14 years age group, which corresponds to the period of the closure of physis of the triradiate cartilage in the acetabulum and the initial age for the indication of a specific surgery (the Bernese periacetabular osteotomy), accounted for 12.8% of total hospitalization costs (R$ 1.652.459,56). The white race was the most frequent (45.69%), although in many cases (35.84%) the race/color was omitted from the AIH record.

**Table 2:** Cases per type of treatment in Group C

Characteristics of the treatments		n (%)	Nominal cost	Mean nominal cost per case	Updated cost *	Mean cost per updated case*
Year of treatment						
	2008	821 (9.56	R$ 1.139.338,95	R$ 1.387,75	R$ 2.219.184,38	R$ 2.703,03
	2009	762 (8.87	R$ 1.008.125,10	R$ 1.323,00	R$ 1.854.209,96	R$ 2.433,35
	2010	839 (9.76	R$ 1.249.921,07	R$ 1.489,77	R$ 2.203.946,90	R$ 2.626,87
	2011	885 (10.30	R$ 1.303.097,53	R$ 1.472,43	R$ 2.169.494,19	R$ 2.451,41
	2012	764 (8.89)	R$ 1.067.892,96	R$ 1.397,77	R$ 1.669.397,21	R$ 2.185,07
	2013	936 (10.89	R$ 1.441.740,47	R$ 1.540,32	R$ 2.129.458,69	R$ 2.275,06
	2014	1.001 (11.65	R$ 1.491.456,30	R$ 1.489,97	R$ 1.921.948,05	R$ 1.920,03
	2015	918 (10.68)	R$ 1.523.182,27	R$ 1.659,24	R$ 1.996.248,46	R$ 2.174,56
	2016	814 (9.47	R$ 1.276.159,86	R$ 1.567,76	R$ 1.511.255,48	R$ 1.856,58
	2017	852 (9.92	R$ 1.389.073,85	R$ 1.630,37	R$ 1.448.942,93	R$ 1.700,64
	Total	8,592 (100.00	R$ 12.889.988,36	R$ 1.500,23	R$ 19.124.086,25	R$ 2.225,80
Race/skin color						
	White	3,926 (45.69	R$ 5.676.559,46	R$ 1.445,89	-	-
	Brown	1,394 (16.22	R$ 2.181.762,60	R$ 1.565,11	-	-
	Black	156 (1.82)	R$ 264.910,11	R$ 1.698,14	-	-
	Yellow	31 (0.36	R$ 48.295,39	R$ 1.557,92	-	-
	Indigenous	6 (0.07	R$ 6.333,45	R$ 1.055,58	-	-
	Not informed	3,079 (35.84)	R$ 4.712.127,35	R$ 1.530,41	-	-
	Total	8,592 (100.00	R$ 12.889.988,36	R$ 1.500,23	R$ 19.124.086,25	R$ 2.225,80
Age range, year						
	<1	975 (11.35	R$ 875.920,02	R$ 898,38	-	-
	1-4	3,240 (37.7	R$ 4.915.685,74	R$ 1.517,19	-	-
	5-9	1,859 (21.64	R$ 3.270.669,77	R$ 1.759,37	-	-
	10-14	1,349 (15.70	R$ 2.175.253,27	R$ 1.612,49	-	-
	15-19	490 (5.70)	R$ 697.117,22	R$ 1.422,69	-	-
	20-24	197 (2.29	R$ 258.793,96	R$ 1.313,67	-	-
	25-29	192 (2.23	R$ 288.284,58	R$ 1.501,48	-	-
	30-34	147 (1.71)	R$ 206.147,46	R$ 1.402,36	-	-
	35-39	143 (1.66	R$ 202.116,34	R$ 1.413,40	-	-
	Total	8,592 (100.00	R$ 12.889.988,36	R$ 1.500,23	R$ 19.124.086,25	R$ 2.225,80
Sex						
	Male	3,049 (35.49	R$ 4.728.067,76	R$ 1.550,69	-	-
	Female	5,543 (64.5	R$ 8.161.920,60	R$ 1.472,47	-	-
	Total	8,592 (100.00	R$ 12.889.988,36	R$ 1.500,23	R$ 19.124.086,25	R$ 2.225,80

Source: prepared by the author based on the data from the Information Technology Department of the Brazilian Public Health System.

Brasil. Ministério da Saúde. Departamento de Informática do Sistema Único de Saúde (DATASUS). Transformação digital para o SUS. Brasília (DF): DATASUS; ©2008 [citado 2020 Abr 24]. Disponível em: http://www2.datasus.gov.br/DATASUS/index.php?area=0901&item=1&acao=25^(12)^

* Values updated by the annual Broad Consumer Price Index (IPCA).

**Figure 1:**
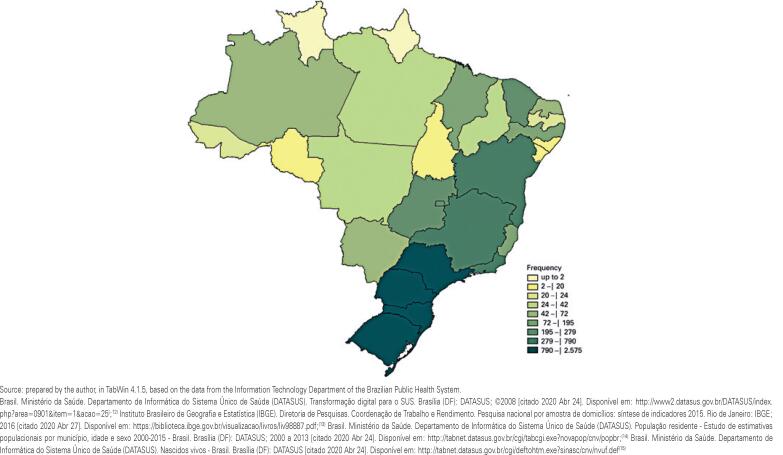
Treatments performed per state, according to the state of treatment in Group C

The map in figure 1 shows the absolute frequency of treatments performed per state, according to the place of performance in Group C.

**Figure 2:**
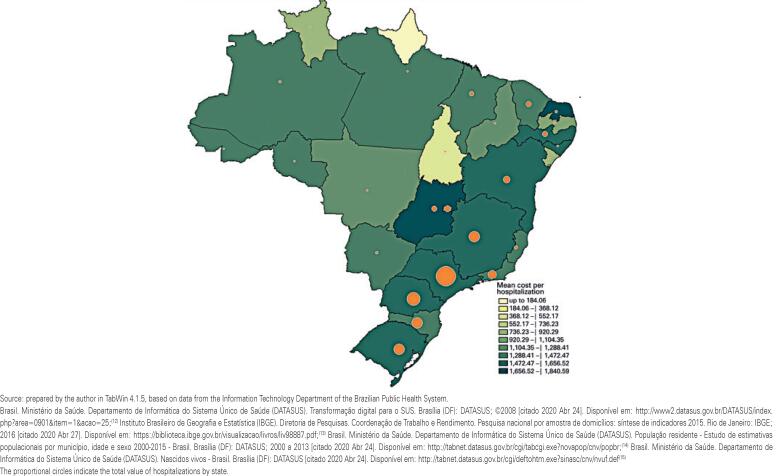
Mean cost per treatment, per state, where the treatment was performed, in Group C

Figure 2 demonstrates the mean cost per treatment in each state of treatment surgery in Group C, and the proportional circles indicate the total value of hospitalizations in each of them. Interestingly, there was a low correlation between the costs per hospitalization and the frequency of treatment per state (R^2^=0.32); some states with low frequency of treatment had high mean hospitalization costs, such as Rio Grande do Norte and Goiás.

During the study period, the rate of surgical treatment per 1,000 live births was, on average, 0.29 per year in Brazil. Regionally, the highest rate of cases performed and originated in the South Region (0.72/1,000 live births, for both rates) and the lowest in the North (0.05 and 0.09/1,000 live births, respectively), as detailed on table 3.

**Table 3:** Rate of cases per one thousand live births, according to region of treatment or of residence of patients

Region of Service	Live births	Cases treated (state of treatment)	Cases originated (state of residence)	Rate of cases performed/1,000 live births)	Rate of cases originated/1,000 live births)
North	3,128,990	172	286	0.054969815	0.091403296
Northeast	8,396,727	1,036	1,032	0.123381408	0.122905032
Southeast	11,481,112	4,010	3,967	0.349269304	0.345524022
South	3,845,176	2,801	2,803	0.7284452	0.728965332
Mid-West	2,327,510	573	504	0.246185838	0.216540423
Total	29,179,515	8,592	8,592	0.294453146	0.294453146

Source: prepared by the author, based on data from Information Technology Department of the Brazilian Public Health System.

Brasil. Ministério da Saúde. Departamento de Informática do Sistema Único de Saúde (DATASUS). Transformação digital para o SUS. Brasília (DF): DATASUS; ©2008 [citado 2020 Abr 24]. Disponível em: http://www2.datasus.gov.br/DATASUS/index.php?area=0901&item=1&acao=25;^(12)^ Instituto Brasileiro de Geografia e Estatística (IBGE). Diretoria de Pesquisas. Coordenação de Trabalho e Rendimento. Pesquisa nacional por amostra de domicílios: síntese de indicadores 2015. Rio de Janeiro: IBGE; 2016 [citado 2020 Abr 27]. Disponível em: https://biblioteca.ibge.gov.br/visualizacao/livros/liv98887.pdf;^(13)^ Brasil. Ministério da Saúde. Departamento de Informática do Sistema Único de Saúde (DATASUS). População residente - Estudo de estimativas populacionais por município, idade e sexo 2000-2015 - Brasil. Brasília (DF): DATASUS; 2000 a 2013 [citado 2020 Abr 24]. Disponível em: http://tabnet.datasus.gov.br/cgi/tabcgi.exe?novapop/cnv/popbr;^(14)^ Brasil. Ministério da Saúde. Departamento de Informática do Sistema Único de Saúde (DATASUS). Nascidos vivos - Brasil. Brasília (DF): DATASUS [citado 2020 Abr 24]. Disponível em: http://tabnet.datasus.gov.br/cgi/deftohtm.exe?sinasc/cnv/nvuf.def^(15)^

A correlation analysis by state showed the factors statistically most associated with the rate of surgical cases of DDH per thousand live births in Group C by state of residence were the proportion of white inhabitants (R²=0.90; p<0.001), the Gini index (R²=-0.80, p<0.001), the proportion of municipalities with high or very high Human Development Index (HDI) (R²=0.69, p<0.001), the number of specialists from SBQ and SBOP (R²=0.75, p<0.001), and the *per capita* income (R²=0.52, p<0.001).

When the association between the same factors and the rate of surgical cases of DDH per one thousand live births was explored in Group C by state, the correlation with white race (R²=0.88) and Gini index (R²=-0.68) decreased. The factors related to care and economic status increased (proportion of municipalities with high or very high HDI, with R²=0.79 and p<0.001; number of SBQ and SBOP specialists, with R²=0.82 and p<0.001); and *per capita* income, with R²=0.68 and p<0.001) (Table 4).

**Table 4:** Rate of cases per one thousand live births, by states and associated factors

State	Group C/1,000 live births per state of treatment	Group C/1,000 live births per state of residence	Resident population*	Population aged less than 40 years*(%)	Hospitals/100 thousand inhabitants*(CNES)	Specialist/1 million inhabitants	Proportion of white inhabitants* (PNAD)	Municipalities with high and very high HDI (>0.7) (%)	Physicians/1,000 inhabitants* (Brazilian Medical Census)	Man/woman* (projection of the population)	Gini index (IBGE census, 2010)	Per capita* income (PNAD)
Acre	0.137	0.160	803,513	76.35	3.11	2.49	0.16	4.54	1.13	1.02	0.64	752.00
Alagoas	0.038	0.042	3,340,932	70.64	2.60	1.20	0.23	0.98	1.28	0.95	0.63	598.00
Amapá	0.000	0.037	766,679	77.13	1.70	2.61	0.23	12.50	1.01	1.02	0.62	840.00
Amazonas	0.094	0.103	3,938,336	76.05	3.00	1.27	0.18	1.61	1.15	1.02	0.67	753.00
Bahia	0.146	0.149	15,203,934	67.42	4.73	3.09	0.19	1.92	1.26	0.98	0.63	736.00
Ceará	0.163	0.164	8,904,459	68.25	3.57	2.25	0.29	2.17	1.26	0.96	0.62	681.00
Distrito Federal	0.494	0.193	2,914,830	67.30	2.50	7.55	0.39	100.00	4.28	0.90	0.64	2.254.00
Espírito Santo	0.243	0.254	3,929,911	64.07	3.56	4.07	0.40	39.74	2.24	1.00	0.57	1.074.00
Goiás	0.245	0.324	6,610,681	65.93	7.12	5.60	0.37	46.30	1.83	1.00	0.56	1.078.00
Maranhão	0.165	0.135	6,904,241	73.93	3.88	0.43	0.18	1.84	0.79	0.98	0.63	509.00
Mato Grosso	0.078	0.133	3,265,486	67.72	5.94	2.45	0.33	34.75	1.42	1.05	0.57	1.053.00
Mato Grosso do Sul	0.126	0.143	2,651,235	65.98	5.17	4.53	0.43	34.61	1.85	1.01	0.57	1.044.00
Minas Gerais	0.305	0.347	20,869,101	62.37	3.93	5.70	0.42	26.73	2.15	0.99	0.56	1.128.00
Pará	0.030	0.064	8,206,923	73.41	3.22	1.71	0.18	2.09	0.91	1.03	0.63	671.00
Paraíba	0.040	0.061	3,972,202	67.00	4.51	1.51	0.36	2.24	1.51	0.94	0.61	774.00
Paraná	0.759	0.750	11,163,018	62.07	5.28	8.87	0.70	59.65	1.96	0.98	0.54	1.241.00
Pernambuco	0.138	0.133	9,345,173	67.14	3.12	2.14	0.31	2.70	1.64	0.94	0.64	825.00
Piauí	0.068	0.076	3,204,028	68.93	4.09	1.25	0.19	0.89	1.17	0.96	0.62	728.00
Rio de Janeiro	0.231	0.211	16,550,024	59.16	4.02	5.92	0.45	63.04	3.75	0.94	0.61	1.284.00
Rio Grande do Norte	0.095	0.114	3,442,175	67.00	3.86	2.03	0.38	2.39	1.50	0.97	0.61	819.00
Rio Grande do Sul	0.580	0.586	11,247,972	58.26	3.71	5.25	0.82	63.10	2.46	0.96	0.55	1.434.00
Rondônia	0.044	0.148	1,768,204	70.33	5.15	1.13	0.28	13.46	1.32	1.04	0.57	823.00
Roraima	0.019	0.066	505,665	76.42	2.37	1.98	0.21	6.66	1.49	1.05	0.64	1.008.00
Santa Catarina	0.905	0.914	6,819,190	62.30	4.36	6.89	0.86	79.18	2.07	1.01	0.49	1.368.00
São Paulo	0.420	0.402	44,396,484	61.27	3.24	8.31	0.63	90.39	2.70	0.97	0.58	1.482.00
Sergipe	0.031	0.039	2,242,937	69.57	3.17	3.12	0.22	1.33	1.54	0.96	0.63	782.00
Tocantins	0.082	0.152	1,515,126	71.49	5.35	1.98	0.21	7.19	1.51	1.03	0.61	816.00
Correlation with the treatment rate^†^	1.00	0.95	0.36	-0.65	0.15	0.82	0.88	0.79	0.54	-0.24	-0.68	0.68
p value^‡^	-	0.000	0.064	0.000	0.452	0.000	0.000	0.000	0.004	0.237	0.000	0.000
Correlation with the residence rate^†^	0.95	1.00	0.36	-0.65	0.28	0.75	0.90	0.69	0.36	-0.04	-0.80	0.52
p value^‡^	0.00	-	0.359	0.000	0.150	0.000	0.000	0.000	0.061	0.849	0.000	0.006

Source: prepared by the author based on data from the Information Technology Department of the Brazilian Public Health System.

Brasil.Ministério da Saúde. Departamento de Informática do Sistema Único de Saúde (DATASUS). Transformação digital para o SUS. Brasília (DF): DATASUS; ©2008 [citado 2020 Abr 24]. Disponível em: http://www2.datasus.gov.br/DATASUS/index.php?area=0901&item=1&acao=25;^(12)^ Instituto Brasileiro de Geografia e Estatística (IBGE). Diretoria de Pesquisas. Coordenação de Trabalho e Rendimento. Pesquisa nacional por amostra de domicílios: síntese de indicadores 2015. Rio de Janeiro: IBGE; 2016 [citado 2020 Abr 27]. Disponível em: https://biblioteca.ibge.gov.br/visualizacao/livros/liv98887.pdf;^(13)^ Brasil. Ministério da Saúde. Departamento de Informática do Sistema Único de Saúde (DATASUS). População residente - Estudo de estimativas populacionais por município, idade e sexo 2000-2015 - Brasil. Brasília (DF): DATASUS; 2000 a 2013 [citado 2020 Abr 24]. Disponível em: http://tabnet.datasus.gov.br/cgi/tabcgi.exe?novapop/cnv/popbr;^(14)^ Brasil. Ministério da Saúde. Departamento de Informática do Sistema Único de Saúde (DATASUS). Nascidos vivos - Brasil. Brasília (DF): DATASUS [citado 2020 Abr 24]. Disponível em: http://tabnet.datasus.gov.br/cgi/deftohtm.exe?sinasc/cnv/nvuf.def;^(15)^ Departamento de Medicina Preventiva. Faculdade de Medicina da Universidade de São Paulo (FMUSP). Conselho Regional de Medicina da Estado de São Paulo (CREMESP). Conselho Federal de Medicina (CFM). Demografia médica no Brasil 2015. São Paulo: FMUSP, CREMESP, CFM; 2015 [citado 2020 Abr 24]. Disponível em: http://www.usp.br/agen/wp-content/uploads/DemografiaMedica30nov20153.pdf;^(16)^ Sociedade Brasileira de Quadril (SBQ). Busca de especialistas. São Paulo: SBQ; 2021 [citado 2020 Fev 24]. Disponível em: https://www.sbquadril.org.br/paciente/buscarespecialista/;^(17)^ Sociedade Brasileira de Ortopedia Pediátrica (SBOP). Institucional/Busca de Especialistas. São Paulo: SBOP; 2021 [citado 2020 Abr 27]. Disponível em: https://www.sbop.org.br/encontre-um-especialista^(18)^

* In 2015;

^†^ Pearson’s correlation;

^‡^ significance (two-tailed).

CNES: *Cadastro Nacional de Estabelecimentos de Saúde*; PNAD: *Pesquisa Nacional por Amostra de Domicílios*; HDI: Human Development Index; IBGE: *Instituto Brasileiro de Geografia e Estatística.*

**Figure 3:**
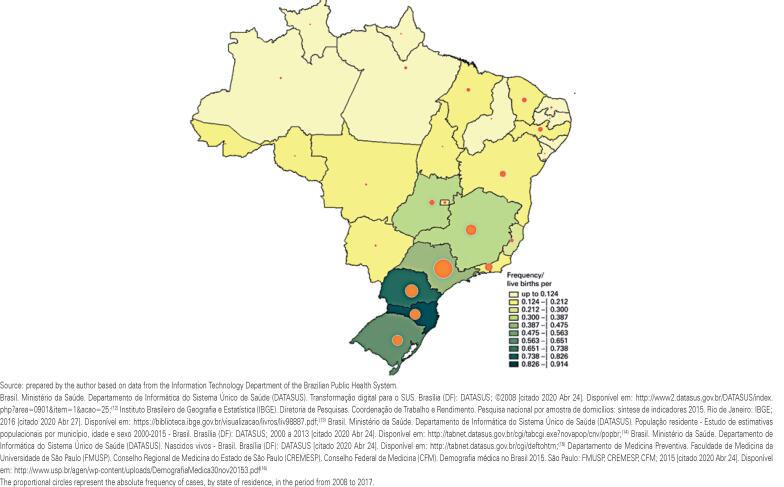
Rate of cases per one thousand live births and absolute frequencies by state of residence in Group C. The colors of the map represent the rates of surgeries for developmental dysplasia of the hip in Group C per one thousand live births by state of residence of the patients, as follows

The rate of treatments per one thousand live births (by state of residence) is shown in figure 3. The proportional circles indicate absolute frequency of treatments, according to residence of patient. The highest rate was found in the State of Santa Catarina with 0.914 cases per one thousand live births, and the lowest in Amapá, with 0.037 cases per one thousand live births.

**Figure 4:**
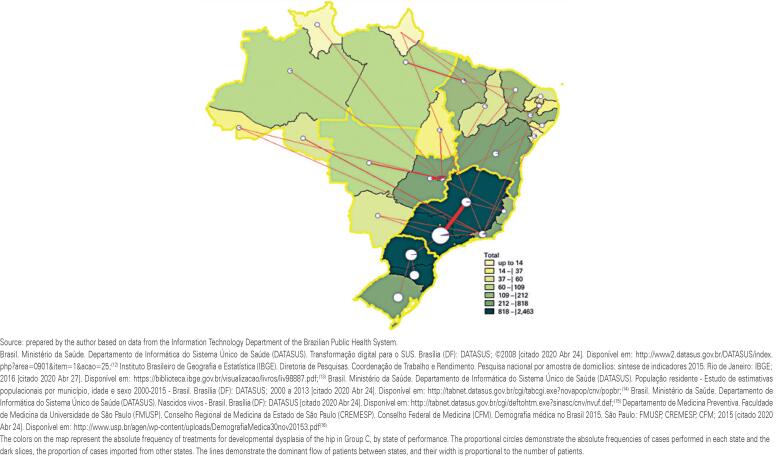
Flow of cases in Group C, according to the state of residence and the state of treatment. For simplification of the image, only the dominant flows are shown

Figure 4 shows the geographical distribution of cases treated in Group C in absolute numbers (by states where surgery was performed) and the flow of patients between states and regions. It is possible to observe that the Southeast Region absorbed the largest number of patients from other regions, and that proportionally, the states of Rio de Janeiro and Maranhão, as well as the Federal District, treated the most patients from other states. We also identified several flows that crossed the boundaries of large geographic regions. Furthermore, 333 case flows between states were registered in the period, the largest between Minas Gerais and São Paulo (85 cases) and between Goiás and the Federal District (69 cases).

## DISCUSSION

In some countries, the prevalence of surgical treatment for DDH has been reported to range from 0.7 to 1.3 per 1,000 live births.^(19)^ This value seems to be influenced by the screening strategy to identify early cases (with or without ultrasonography), by hereditary and racial factors.^(19,20)^ A study evaluating the prevalence of surgical interventions in DDH (acetabuloplasty, osteotomy, triple osteotomy, periacetabular osteotomy) in patients born in Austria, Germany, and the Czech Republic from 1992 to 2008 reported a 46% reduction over a 16-year period.^(19)^ In that study, the routine use of screening ultrasound examination was implicated in the decreased need for this type of surgical approach.^(19)^ A rate of 0.29 cases of operated DDH per 1,000 live births was found in our country (ranging from 0.72 to 0.05, depending on the region), which is lower than that reported in the literature.^(19)^ This lower frequency of treatment deserves discussion.

First, this study did not include surgeries performed in the private services, which may underestimate the real frequency of treatments performed in Brazil, where about 25.6% of population (2015 data) had access to health insurance plans. The heterogeneous geographic distribution seen in this study confirms the hypothesis initially postulated by Puech,^(11)^ that racial and demographic factors impact on epidemiology of the disease. The Southern Region of the country, which has a large proportion of European migrants, had a treatment rate for DDH more than twice the national average and 15-fold higher than that of the Northern Region. In fact, a nationwide genetic study found a prevalence of up to 85% of European ancestry in some municipalities of that region.^(21)^ The strong correlation between the incidence of surgeries and the proportion of white individuals in the states verified in this study, reinforces the impression that part of this correlation is related to hereditary factors.

However, other factors seem to influence this distribution. Since the study did not directly measure the prevalence of the disease, but that of treatment of a complication of the disease, findings may be related to inequities in access to diagnosis and treatment. Regions with smaller healthcare facilities (lower rate of specialists) and lower socioeconomic indices (income *per capita* and HDI) have lower rates of cases. In addition, the Southern and Southeastern states received the greatest flow of patients, which increased the polarization of case performance in these centers. In fact, this finding reflects a violation of the SUS principles, whose hierarchical organization presupposes that this type of treatment, in most cases, could be offered at the state level or at most, regional level. The logic of treatment away from home overloads the large centers, generates indirect costs, hinders regional development, discourages the establishment of specialized professionals in inland, and hurts the principle of decentralization of the SUS. In addition, it generates suffering for families who need to travel, in some cases, thousands of kilometers with their small children to receive the appropriate treatment.^(22)^

The literature indicates a four to eight times higher prevalence of DDH in women.^(3,5,23)^ In this study, females corresponded to 67.8% of cases, confirming their predominance in Brazil. This contrasts to a series of cases previously published in Brazil, which reported a higher prevalence in males.^(10)^

When analyzing the results obtained in relation to the racial distribution of DDH surgery, a clear predominance of the white race (42.06%) is observed. This has been reported in other studies in our country, although with different proportions. In a national study, a predominance of the white race was found with a frequency of 81% of cases.^(10)^ This difference may be related to the incomplete filling out of AIHs in Brazil, since in 41.6% of cases included in this study, there was no information regarding the patient’s race.

Surgical treatment of DDH is most prevalent among children aged 1 to 4 years in our setting. This is compatible with the most common age range for the indication of surgical treatment of neglected DDH.^(9)^ Considering the reports in the literature that for every 100 cases of DDH, 19 progress to surgery,^(19)^ it is possible to estimate a Brazilian incidence of DDH of 1.52 per one thousand live births (ranging from 3.78 to 0.26 per one thousand live births per region, the highest in Santa Catarina). This means that the estimated incidence of DDH cases in Brazil is lower than that of Mediterranean and Eastern European countries, Japan, Australia, and New Zealand, and higher than some African and Western European countries, besides maintaining similarity with some states in the United States and other South American countries, such as Chile.^(24)^

The differences found in the frequencies of treatments in different groups (search strategies), although revealing different specificities of each search parameter, also led us to discuss the importance of the correct completion of the AIH. Since the information in this study derives from secondary data, factors that influence the filling out and recording of AIHs can interfere with the results. Among these factors, the literature mentions the completeness and correctness of the information filled out by the physician; difficulty in deciphering the physician’s handwriting; lack of training, and lack of knowledge of the coding rules on the part of hospital employees.^(25)^ An example of this situation was observed in this study, in which the race of the patients was not documented in more than 40% of cases.

There are limitations to this study. This is an ecological study, there is no claim to show causal relations, but simply to analyze associated factors. However, by exploring a robust nationwide database, this study brings to light new information regarding the Brazilian epidemiology of DDH and its complications, in which individual impact on the health of patients and their families can be devastating. A potential bias is related to the quality of the original AIH. The use of three different search strategies (Groups A, B, and C) allowed us to select data more specific to the research object. However, it is not possible to identify all cases of completion errors, omissions, or fraud. In addition to the original information contained in this study, it can help make physicians aware of the importance of feeding the official databases, filling out the HIA correctly, and providing reliable epidemiological information.

## CONCLUSION

The frequent surgical treatment of developmental dysplasia of the hip in Brazil generates relevant costs. It is distributed heterogeneously and unevenly throughout the Public Health System. Southern states have the highest incidence of cases. Racial and socioeconomic factors are associated with this distribution. There was a small temporal variation in the incidence of operated cases in the period of a decade.
