# Supplementary material for: Epidemiology and costs of surgical treatment of developmental dysplasia of hip in the Brazilian Public Health System in a decade
Source: Einstein (Sao Paulo). 2021 Nov 24;19:eGS5625. doi: 10.31744/einstein_journal/2021GS5625 (PMC8664290; doi:10.31744/einstein_journal/2021GS5625)
Supplement: Translation

# Translation: Epidemiologia e custos do tratamento cirúrgico da displasia do desenvolvimento do quadril no Sistema Único de Saúde em uma década

**Authors:** Bruno Gonçalves Schröder e Souza, Bruno Marinho Coelho Vasconcelos, Higor Pereira Pujoni, Mário Círio Nogueira, Valdeci Manoel de Oliveira, Alfredo Chaoubah

**Affiliations:** 1 Juiz de Fora MG Brasil Programa de Pós-Graduação em Saúde Coletiva, Universidade Federal de Juiz de Fora, Juiz de Fora, MG, Brasil.; 2 Juiz de Fora MG Brasil Núcleo de Pesquisa em Ortopedia e Traumatologia, Hospital e Maternidade Therezinha de Jesus, Juiz de Fora, MG, Brasil.

**Keywords:** Displasia do desenvolvimento do quadril, Prevalência, Sistema Único de Saúde, Análise espacial, Estudos de séries temporais, Custos e análise de custo, Brasil

## Abstract

**Objetivo:**

Descrever e analisar a epidemiologia e os custos do tratamento cirúrgico da displasia do quadril no Sistema Único de Saúde.

**Métodos:**

Estudo ecológico analítico da série temporal e distribuição geográfica dos casos de tratamento cirúrgico da displasia no Brasil. Foram avaliadas: frequências, taxas de casos e fatores de associação. Taxas de correlação, mapas de frequência e fluxos são apresentados e discutidos.

**Resultados:**

No período, foram internados 14.584 pacientes com displasia segundo o Departamento de Informática do Sistema Único de Saúde. Os pacientes receberam tratamento hospitalar específico para displasia em 8.592 casos (custo médio de R$ 2.225,50 por internação), com custo de R$ 19.124.086,25 (valores atualizados). Nesse grupo, o tempo médio de internação foi de 4,41 dias (desvio-padrão de 2,39 dias) e a mortalidade foi de 0,046%. Idade de 1 a 4 anos (37,7%), sexo feminino (64,5%) e raça branca (46%) foram as mais frequentes. Maiores taxas de especialistas (R²=0,82; p<0,001), maior proporção de municípios com desenvolvimento humano alto/muito alto (R²=0,79; p<0,001) e maior renda *per capita* (R²=0,68; p<0,001) foram relacionadas a maiores taxas de tratamentos realizados por mil nascidos vivos (por estado do tratamento). O fator mais relacionado à taxa de tratamentos realizados por mil nascidos vivos (conforme o estado de residência) foi a raça branca (R²=0,90; p<0,001). Estados do Sul tiveram as maiores taxas de tratamentos (por estado de residência, com taxa de 0,73/1.000), e os do Sudeste a maior frequência absoluta de casos (46,7%) e o maior influxo de pacientes.

**Conclusão:**

O tratamento cirúrgico para displasia do quadril no Brasil é frequente, gera custos relevantes e é distribuído de forma heterogênea e desigual no Sistema Único de Saúde. Estados do Sul tem a maior incidência de casos, e fatores raciais e socioeconômicos estão associados. Não houve grande variação na frequência de casos no período.

## INTRODUÇÃO

A displasia do desenvolvimento do quadril (DDQ) é uma condição em que a cabeça femoral tem uma relação anormal com o acetábulo e cuja gravidade varia de casos de instabilidade ao nascimento até luxação.^(1)^ Na ausência de diagnóstico ou no diagnóstico tardio (após os primeiros meses de vida), o tratamento se torna mais complexo, a morbidade aumenta e as chances de normalidade do desenvolvimento do quadril diminuem.^(2,3)^ Trata-se, então, de fator de risco estabelecido para a coxartrose precoce (antes dos 50 anos de idade), sendo uma afecção de importante impacto econômico e social.^(4)^

Em estudo recente, realizado em hospital de ensino no Brasil, foi detectada importante falha de conhecimento dos pediatras e residentes de pediatria, que são os responsáveis pela triagem clínica e o consequente encaminhamento precoce dos pacientes para o tratamento.^(5)^ Em outros países, estratégias como a realização sistemática de ultrassonografia foram efetivadas para evitar que esses casos deixassem de ser identificados no momento ideal.^(6)^ No entanto, a custo-efetividade dessa estratégia foi questionada, e esse tipo de triagem só parece ser adequado em locais onde a prevalência de cirurgias seja elevada.^(7,8)^ No Brasil, essa prevalência ainda não parece ter sido estudada.

De fato, os dados epidemiológicos dessa doença variam conforme a região geográfica.^(9)^ A incidência da DDQ é estimada de 1,5 a 20 por mil nascidos vivos, sendo quatro a oito vezes mais prevalente em mulheres.^(3,5)^ Existe uma variabilidade significativa na incidência entre grupos raciais na mesma localização geográfica. A incidência da instabilidade clínica do quadril neonatal ao nascimento varia, por exemplo, de 0,4 por mil, nos africanos, a 61,7 por mil, nos caucasianos poloneses.^(9)^

No Brasil, Puech foi o pioneiro em discutir a epidemiologia da DDQ. Ele argumentou que, em nosso país, no início do século 20, havia baixa frequência dessa afecção, o que se modificou em decorrência de correntes migratórias, principalmente as provenientes da Europa.^(10,11)^ No entanto, não foram identificados estudos na última década que tenham explorado a epidemiologia da DDQ e seu tratamento.

A hipótese nula deste estudo é que a incidência do tratamento cirúrgico da DDQ no Sistema Único de Saúde (SUS) tenha frequência semelhante a de outros países, não varie ao longo dos anos e seja distribuída de forma não uniforme nas unidades da federação.

## OBJETIVO

Descrever e analisar a epidemiologia e os custos do tratamento cirúrgico da displasia do quadril no Sistema Único de Saúde.

## MÉTODOS

Trata-se de estudo ecológico analítico, que avaliou a série temporal e a distribuição espacial dos casos de tratamento cirúrgico da DDQ no SUS no período de uma década (entre 2008 a 2017), com coleta de dados secundários, baseado na análise dos dados do Departamento de Informática do Sistema Único de Saúde (DATASUS).^(12)^

Conforme a resolução 510 de 2016, da Comissão Nacional de Ética em Pesquisa (Conep), pesquisas com dados secundários de bancos de dados públicos de acesso irrestrito estão dispensadas de obter parecer do Comitê de Ética em Pesquisa (CEP). Esta pesquisa foi desenvolvida como parte do Programa de Pós-Graduação em Saúde Coletiva da Universidade Federal de Juiz de Fora (UFJF) e do Núcleo de Pesquisa em Ortopedia e Traumatologia do Hospital e Maternidade Therezinha de Jesus, em Juiz de Fora (MG).

Os arquivos de dados foram obtidos na plataforma do DATASUS, incluindo toda a base de dados do Sistema de Informações Hospitalares do SUS (Sistema SIHSUS),^(12)^ a base populacional (com dados da estimativa populacional brasileira de 2015),^(13)^ inquéritos e pesquisas (Pesquisa Nacional por Amostra de Domicílios – PNAD)^(14)^ e a base do Sistema de Informações sobre Nascidos Vivos (SINASC),^(15)^ sendo tabulados e analisados no programa TabWin 4.1.5 e apresentados em forma de gráficos, tabelas e mapas. Paralelamente, utilizou-se a pesquisa de demografia médica brasileira de 2015^(16)^ e os registros de especialistas da Sociedade Brasileira de Quadril (SBQ) e da Sociedade Brasileira de Ortopedia Pediátrica (SBOP), que contêm os dados quantitativos com o número de especialistas nessas cirurgias, por unidade geográfica.^(17,18)^

Foram incluídos no estudo todos os dados das Autorização de Internação Hospitalar (AIH) realizadas no SUS, no período de janeiro de 2008 a dezembro de 2017.

Dentre as AIH inclusas no trabalho, foram selecionados casos em que houve o tratamento tardio da DDQ. Para tal, foram feitas diferentes seleções nos parâmetros do programa TabWin, gerando os Grupos A, B e C.

O Grupo A incluiu todos os pacientes internados cujo campo diagnóstico principal contivesse o código Q65 da Classificação Internacional de Doenças e Problemas Relacionados à Saúde (CID-10), que se refere à displasia do desenvolvimento do quadril. Esse parâmetro objetivou promover uma busca ampla, com boa sensibilidade para condições relacionadas ao diagnóstico de displasia, embora permitisse a inclusão de vários casos não necessariamente relacionados ao tratamento hospitalar tardio (cirúrgico) da DDQ.

No Grupo B, foi incluído um filtro adicional para restringir os casos obtidos na seleção do Grupo A, melhorando a especificidade da busca. O filtro consistiu de 11 tratamentos cirúrgicos típicos do tratamento tardio da DDQ, cujos códigos do SUS são: 0408040327, 0408060190, 0408040181, 0408040343, 0415010012, 0408040157, 0408040173, 0408040220, 0415020034, 0408040165 e 0415020069, conforme a Tabela de Procedimentos, Medicamentos, Órteses, Próteses e Materiais Especiais do SUS.

No Grupo C, para remover os casos não relacionados a cirurgias preservadoras de quadril, foram excluídos todos os casos de pacientes com idade igual ou superior a 40 anos de idade.

Os casos incluídos no Grupo C fomaram a amostra final da pesquisa, a qual serviu para todas as comparações e análises. As variáveis de desfecho foram as frequências absolutas anuais e taxas de tratamentos realizados por mil nascidos vivos no período. Os dados foram tabulados e apresentados por unidade federativa e região geográfica de realização do tratamento e de residência do paciente. Também foram avaliadas as seguintes variáveis para a exploração de associações: idade do paciente, sexo, raça, variáveis demográficas (taxa de nascidos vivos, população residente e renda domiciliar per capita), além das assistenciais (razão de hospitais com internação por 100 mil habitantes, razão de faculdades de medicina por 100 mil habitantes), razão de especialistas da SBQ e SBOP por 1 milhão de habitantes e custos hospitalares totais e por internação.

Os dados foram apresentados como estatísticas descritivas por ano (taxas de incidência, percentagens, médias e desvios-padrão – DP). Para estudo de associações, foi calculada a taxa de tratamentos cirúrgicos realizados por mil nascidos vivos por unidade da federação e utilizadas tabelas de correlações bivariáveis, usando o coeficiente de correlação de Pearson, com teste de significância bicaudal e p<0,05. Os testes estatísticos foram realizados no programa (SPSS), versão 21.

A distribuição geográfica dos tratamentos é apresentada por meio de mapas gerados a partir dos dados plotados no programa TabWin (versão 4.1.5).

## RESULTADOS

No período de janeiro de 2008 a dezembro de 2017, foram registradas, no Brasil, 14.584 internações de pacientes com diagnóstico principal de displasia de quadril (CID-10 Q65) no SUS (Grupo A). Foram encontrados 68 diferentes tipos de tratamentos realizados dentre esses pacientes, muitos dos quais não guardavam relação com o objeto deste estudo (por exemplo: parto cesáreo, tratamento conservador de fraturas, tratamento cirúrgico de politraumatizados, entre outros). A tabela 1 mostra a frequência de todos os tratamentos realizados no Grupo A e a frequência encontrada no Grupo B, após a aplicação do filtro de tratamentos.

**Tabela 1:** Casos por tipo de tratamento no Grupo A

Tratamento realizado identificado por código e nome	Grupo A n (%)	Incluso no Grupo B
0408040327 – Tratamento cirúrgico de luxação coxofemoral congênita	2.464 (16,90)	Sim
0408060190 – Osteotomia de ossos longos exceto da mão e do pé	1.149 (7,88)	Sim
0408040181 – Redução incruenta de luxação congênita coxofemoral	1.102 (7,56)	Sim
0408040343 – Tratamento cirúrgico de luxação espontânea/ progressiva/ paralítica do quadril	996 (6,83)	Sim
0415010012 – Tratamento com cirurgias múltiplas	867 (5,94)	Sim
0408040157 – Osteotomia da pelve	845 (5,79)	Sim
0408040173 – Redução incruenta com manipulação de luxação espontânea/progressiva do quadril com aplicação de dispositivo ortopédico	721 (4,94)	Sim
0408040220 – Revisão cirúrgica de luxação coxofemoral congênita	475 (3,26)	Sim
0415020034 – Outros tratamentos com cirurgias sequenciais	472 (3,24)	Sim
0408040165 – Reconstrução osteoplástica do quadril	255 (1,75)	Sim
0415020069 – Tratamentos sequenciais em ortopedia	124 (0,85)	Sim
Outros 57 tipos de tratamentos não relacionados ao escopo deste estudo	5.114 (35,06)	Não
Total	14.584 (100)	

Fonte: elaborado pelo autor, com base nos dados do Departamento de Informática do Sistema Único de Saúde.

Brasil. Ministério da Saúde. Departamento de Informática do Sistema Único de Saúde (DATASUS). Transformação digital para o SUS. Brasília (DF): DATASUS; ©2008 [citado 2020 Abr 24]. Disponível em: http://www2.datasus.gov.br/DATASUS/index. php?area=0901&item=1&acao=25^(12)^

Para o Grupo B, foram utilizados CID e código de tratamento como filtros, sendo obtida frequência absoluta de 9.470 casos tratados em uma década. Nesse grupo, foram contabilizados 22 óbitos (mortalidade de 0,23%), e a permanência hospitalar média foi de 5,07 dias (mínimo de 1,5 e máximo de 9,95 dias).

O Grupo C, que incluiu um filtro adicional de idade, representando a amostra de interesse deste estudo (ou seja, de pacientes portadores de displasia, submetidos a tratamentos cirúrgicos de preservação do quadril), contabilizou 8.592 internações. O valor nominal pago pelo SUS em uma década foi de R$ 12.889.988,36. De acordo com correções do Índice de Preços ao Consumidor Amplo (IPCA) para janeiro de 2020, o valor foi de R$ 19.124.086,25 (média de R$ 2.225,50 por internação). Nesse grupo, ocorreram quatro óbitos (mortalidade de 0,046%), com permanência hospitalar média de 4,41 dias (DP=2,39; mínimo de 1,5 e máximo de 10,48 dias).

A tabela 2 mostra a evolução temporal das frequências e os custos de tratamentos no Grupo C. Em média, foram realizados 947 casos por ano (DP=85,80). A linha de tendência do Grupo C mostra um acréscimo menor que 10% no número de tratamentos ao longo da década. Além disso, a tabela 2 também apresenta outros dados de frequência e custo no Grupo C. O sexo feminino foi o mais frequente (64,5% no Grupo C). A média de idade na internação foi de 8,14 anos (DP=7,63 anos). A distribuição de custos por faixa etária seguiu distribuição semelhante à da frequência (R^2^=0,99). A faixa etária acima de 14 anos, que corresponde ao período do fechamento da fise da cartilagem trirradiada no acetábulo e idade inicial para a indicação de uma cirurgia específica (a osteotomia periacetabular bernesa), representou 12,8% dos custos totais com internações (R$ 1.652.459,56). A raça branca foi a mais frequente (45,69%), embora em muitos casos (35,84%) a raça/cor tenha sido omitida do registro da AIH.

**Tabela 2:** Casos por tipo de tratamento no Grupo C

Características dos tratamentos		n (%)	Custo nominal	Custo nominal médio por caso	Custo atualizado*	Custo médio por caso atualizado*
Ano do tratamento						
	2008	821 (9,56)	R$ 1.139.338,95	R$ 1.387,75	R$ 2.219.184,38	R$ 2.703,03
	2009	762 (8,87)	R$ 1.008.125,10	R$ 1.323,00	R$ 1.854.209,96	R$ 2.433,35
	2010	839 (9,76)	R$ 1.249.921,07	R$ 1.489,77	R$ 2.203.946,90	R$ 2.626,87
	2011	885 (10,30)	R$ 1.303.097,53	R$ 1.472,43	R$ 2.169.494,19	R$ 2.451,41
	2012	764 (8,89)	R$ 1.067.892,96	R$ 1.397,77	R$ 1.669.397,21	R$ 2.185,07
	2013	936 (10,89)	R$ 1.441.740,47	R$ 1.540,32	R$ 2.129.458,69	R$ 2.275,06
	2014	1.001 (11,65)	R$ 1.491.456,30	R$ 1.489,97	R$ 1.921.948,05	R$ 1.920,03
	2015	918 (10,68)	R$ 1.523.182,27	R$ 1.659,24	R$ 1.996.248,46	R$ 2.174,56
	2016	814 (9,47)	R$ 1.276.159,86	R$ 1.567,76	R$ 1.511.255,48	R$ 1.856,58
	2017	852 (9,92)	R$ 1.389.073,85	R$ 1.630,37	R$ 1.448.942,93	R$ 1.700,64
	Total	8.592 (100,00)	R$ 12.889.988,36	R$ 1.500,23	R$ 19.124.086,25	R$ 2.225,80
Raça/cor						
	Branca	3.926 (45,69)	R$ 5.676.559,46	R$ 1.445,89	-	-
	Parda	1.394 (16,22)	R$ 2.181.762,60	R$ 1.565,11	-	-
	Preta	156 (1,82)	R$ 264.910,11	R$ 1.698,14	-	-
	Amarela	31 (0,36)	R$ 48.295,39	R$ 1.557,92	-	-
	Indígena	6 (0,07)	R$ 6.333,45	R$ 1.055,58	-	-
	Não informado	3.079 (35,84)	R$ 4.712.127,35	R$ 1.530,41	-	-
	Total	8.592 (100,00)	R$ 12.889.988,36	R$ 1.500,23	R$ 19.124.086,25	R$ 2.225,80
Faixa etária, ano						
	<1	975 (11,35)	R$ 875.920,02	R$ 898,38	-	-
	1-4	3.240 (37,7)	R$ 4.915.685,74	R$ 1.517,19	-	-
	5-9	1.859 (21,64)	R$ 3.270.669,77	R$ 1.759,37	-	-
	10-14	1.349 (15,70)	R$ 2.175.253,27	R$ 1.612,49	-	-
	15-19	490 (5,70)	R$ 697.117,22	R$ 1.422,69	-	-
	20-24	197 (2,29)	R$ 258.793,96	R$ 1.313,67	-	-
	25-29	192 (2,23)	R$ 288.284,58	R$ 1.501,48	-	-
	30-34	147 (1,71)	R$ 206.147,46	R$ 1.402,36	-	-
	35-39	143 (1,66)	R$ 202.116,34	R$ 1.413,40	-	-
	Total	8.592 (100,00)	R$ 12.889.988,36	R$ 1.500,23	R$ 19.124.086,25	R$ 2.225,80
Sexo						
	Masculino	3.049 (35,49)	R$ 4.728.067,76	R$ 1.550,69	-	-
	Feminino	5.543 (64,5)	R$ 8.161.920,60	R$ 1.472,47	-	-
	Total	8.592 (100,00)	R$ 12.889.988,36	R$ 1.500,23	R$ 19.124.086,25	R$ 2.225,80

Fonte: elaborado pelo autor, com base nos dados do Departamento de Informática do Sistema Único de Saúde.

Brasil. Ministério da Saúde. Departamento de Informática do Sistema Único de Saúde (DATASUS). Transformação digital para o SUS. Brasília (DF): DATASUS; ©2008 [citado 2020 Abr 24]. Disponível em: http://www2.datasus.gov.br/DATASUS/index.php?area=0901&item=1&acao=25^(12)^

* Valores atualizados pelo Índice de Preço ao Consumidor Amplo anual.

A figura 1 mostra a frequência absoluta de tratamentos realizados por unidade federativa, segundo o local de realização no Grupo C.

**Figura 1:** Tratamentos realizados por unidade federativa, segundo o local de realização no Grupo C

A figura 2 demonstra o custo médio por tratamento em cada unidade federativa de realização da cirurgia no Grupo C, e os círculos proporcionais indicam o valor total das internações em cada uma delas. Interessantemente foi baixa a correlação entre os custos por internação e a frequência de tratamentos realizados por unidade federativa (R^2^=0,32), e algumas unidades federativas com baixa frequência de tratamentos tiveram elevado custo médio de internação, a exemplo do Rio Grande do Norte e de Goiás.

**Figura 2:** Custo médio por tratamento, por unidade federativa de realização do tratamento, no Grupo C

No período do estudo, a taxa de tratamento cirúrgico por mil nascidos vivos foi, em média, de 0,29 por ano no Brasil. Regionalmente, foi observada a maior taxa de casos realizados e originados na Região Sul (0,72/1.000 nascidos vivos, para ambas as taxas) e a menor na Norte (0,05 e 0,09/1.000 nascidos vivos, respectivamente) conforme detalhado na tabela 3.

**Tabela 3:** Taxa de casos por mil nascidos vivos, conforme região do tratamento ou da residência dos pacientes

Região de atendimento	Nascidos vivos	Casos realizados (local da cirurgia)	Casos originados (residência do paciente)	Taxa de casos realizados/1.000 nascidos vivos	Taxa de casos originados/1.000 nascidos vivos
Norte	3.128.990	172	286	0,054969815	0,091403296
Nordeste	8.396.727	1.036	1.032	0,123381408	0,122905032
Sudeste	11.481.112	4.010	3.967	0,349269304	0,345524022
Sul	3.845.176	2.801	2.803	0,7284452	0,728965332
Centro-Oeste	2.327.510	573	504	0,246185838	0,216540423
Total	29.179.515	8.592	8.592	0,294453146	0,294453146

Fonte: elaborado pelo autor com base nos dados do Departamento de Informática do Sistema Único de Saúde.

Brasil. Ministério da Saúde. Departamento de Informática do Sistema Único de Saúde (DATASUS). Transformação digital para o SUS. Brasília (DF): DATASUS; ©2008 [citado 2020 Abr 24]. Disponível em: http://www2.datasus.gov.br/DATASUS/index.php?area=0901&item=1&acao=25;^(12)^ Instituto Brasileiro de Geografia e Estatística (IBGE). Diretoria de Pesquisas. Coordenação de Trabalho e Rendimento. Pesquisa nacional por amostra de domicílios: síntese de indicadores 2015. Rio de Janeiro: IBGE; 2016 [citado 2020 Abr 27]. Disponível em: https://biblioteca.ibge.gov.br/visualizacao/livros/liv98887.pdf;^(13)^ Brasil. Ministério da Saúde. Departamento de Informática do Sistema Único de Saúde (DATASUS). População residente - Estudo de estimativas populacionais por município, idade e sexo 2000-2015 - Brasil. Brasília (DF): DATASUS; 2000 a 2013 [citado 2020 Abr 24]. Disponível em: http://tabnet.datasus.gov.br/cgi/tabcgi.exe?novapop/cnv/popbr;^(14)^ Brasil. Ministério da Saúde. Departamento de Informática do Sistema Único de Saúde (DATASUS). Nascidos vivos - Brasil. Brasília (DF): DATASUS [citado 2020 Abr 24]. Disponível em: http://tabnet.datasus.gov.br/cgi/deftohtm^(15)^

Em uma análise de correlação por unidade federativa, observa-se que os fatores estatisticamente mais associados à taxa de casos cirúrgicos de DDQ por mil nascidos vivos no Grupo C por unidade federativa de residência foram a proporção de habitantes da raça branca (R²=0,90; p<0,001), o índice de Gini (R²=-0,80; p<0,001), a proporção de municípios com Índice de Desenvolvimento Humano (IDH) alto ou muito alto (R²=0,69; p<0,001), o número de especialistas da SBQ e SBOP (R² =0,75; p<0,001), e a renda *per capita* (R²=0,52; p<0,001).

Quando explorada a associação entre os mesmos fatores e a taxa de casos cirúrgicos de DDQ por mil nascidos vivos no Grupo C por unidade federativa de tratamento, a correlação com a raça branca (R²=0,88) e o índice de Gini (R²=-0,68) diminuíram, e os fatores relacionados à assistência e à situação econômica aumentaram (proporção de municípios com IDH alto ou muito alto, com R²=0,79 e p<0,001); número de especialistas da SBQ e SBOP, com R²=0,82 e p<0,001); e renda *per capita*, com R²= 0,68 e p<0,001) (Tabela 4).

**Tabela 4:** Taxa de casos por mil nascidos vivos, por unidade federativa e fatores associados

UF	Grupo C/1.000 nascidos vivos por UF de atendimento	Grupo C/1.000 nascidos vivos por UF de residência	População residente*	População com menos de 40 anos de idade* (%)	Hospitais/100.000 habitantes* (CNES)	Especialista/1.000.000 habitantes	Proporção de habitantes brancos* (PNAD)	Municípios com IDH alto e muito alto (>0,7) (%)	Médicos/1.000 habitantes* (Censo Médico Brasileiro)	Homem/mulher* (projeção da população)	Índice de Gini (Censo IBGE, 2010)	Renda per capita* (PNAD)
Acre	0,137	0,160	803.513	76,35	3,11	2,49	0,16	4,54	1,13	1,02	0,64	752,00
Alagoas	0,038	0,042	3.340.932	70,64	2,60	1,20	0,23	0,98	1,28	0,95	0,63	598,00
Amapá	0,000	0,037	766.679	77,13	1,70	2,61	0,23	12,50	1,01	1,02	0,62	840,00
Amazonas	0,094	0,103	3.938.336	76,05	3,00	1,27	0,18	1,61	1,15	1,02	0,67	753,00
Bahia	0,146	0,149	15.203.934	67,42	4,73	3,09	0,19	1,92	1,26	0,98	0,63	736,00
Ceará	0,163	0,164	8.904.459	68,25	3,57	2,25	0,29	2,17	1,26	0,96	0,62	681,00
Distrito Federal	0,494	0,193	2.914.830	67,30	2,50	7,55	0,39	100,00	4,28	0,90	0,64	2.254,00
Espírito Santo	0,243	0,254	3.929.911	64,07	3,56	4,07	0,40	39,74	2,24	1,00	0,57	1.074,00
Goiás	0,245	0,324	6.610.681	65,93	7,12	5,60	0,37	46,30	1,83	1,00	0,56	1.078,00
Maranhão	0,165	0,135	6.904.241	73,93	3,88	0,43	0,18	1,84	0,79	0,98	0,63	509,00
Mato Grosso	0,078	0,133	3.265.486	67,72	5,94	2,45	0,33	34,75	1,42	1,05	0,57	1.053,00
Mato Grosso do Sul	0,126	0,143	2.651.235	65,98	5,17	4,53	0,43	34,61	1,85	1,01	0,57	1.044,00
Minas Gerais	0,305	0,347	20.869.101	62,37	3,93	5,70	0,42	26,73	2,15	0,99	0,56	1.128,00
Pará	0,030	0,064	8.206.923	73,41	3,22	1,71	0,18	2,09	0,91	1,03	0,63	671,00
Paraíba	0,040	0,061	3.972.202	67,00	4,51	1,51	0,36	2,24	1,51	0,94	0,61	774,00
Paraná	0,759	0,750	11.163.018	62,07	5,28	8,87	0,70	59,65	1,96	0,98	0,54	1.241,00
Pernambuco	0,138	0,133	9.345.173	67,14	3,12	2,14	0,31	2,70	1,64	0,94	0,64	825,00
Piauí	0,068	0,076	3.204.028	68,93	4,09	1,25	0,19	0,89	1,17	0,96	0,62	728,00
Rio de Janeiro	0,231	0,211	16.550.024	59,16	4,02	5,92	0,45	63,04	3,75	0,94	0,61	1.284,00
Rio Grande do Norte	0,095	0,114	3.442.175	67,00	3,86	2,03	0,38	2,39	1,50	0,97	0,61	819,00
Rio Grande do Sul	0,580	0,586	11.247.972	58,26	3,71	5,25	0,82	63,10	2,46	0,96	0,55	1.434,00
Rondônia	0,044	0,148	1.768.204	70,33	5,15	1,13	0,28	13,46	1,32	1,04	0,57	823,00
Roraima	0,019	0,066	505.665	76,42	2,37	1,98	0,21	6,66	1,49	1,05	0,64	1.008,00
Santa Catarina	0,905	0,914	6.819.190	62,30	4,36	6,89	0,86	79,18	2,07	1,01	0,49	1.368,00
São Paulo	0,420	0,402	44.396.484	61,27	3,24	8,31	0,63	90,39	2,70	0,97	0,58	1.482,00
Sergipe	0,031	0,039	2.242.937	69,57	3,17	3,12	0,22	1,33	1,54	0,96	0,63	782,00
Tocantins	0,082	0,152	1.515.126	71,49	5,35	1,98	0,21	7,19	1,51	1,03	0,61	816,00
Correlação com a taxa de atendimento †	1,00	0,95	0,36	-0,65	0,15	0,82	0,88	0,79	0,54	-0,24	-0,68	0,68
Valor de p‡	-	0,000	0,064	0,000	0,452	0,000	0,000	0,000	0,004	0,237	0,000	0,000
Correlação com a taxa de residência†	0,95	1,00	0,36	-0,65	0,28	0,75	0,90	0,69	0,36	-0,04	-0,80	0,52
Valor de p‡	0,00	-	0,359	0,000	0,150	0,000	0,000	0,000	0,061	0,849	0,000	0,006

Fonte: elaborado pelo autor, com base nos dados do Departamento de Informática do Sistema Único de Saúde.

Brasil. Ministério da Saúde. Departamento de Informática do Sistema Único de Saúde (DATASUS). Transformação digital para o SUS. Brasília (DF): DATASUS; ©2008 [citado 2020 Abr 24]. Disponível em: http://www2.datasus.gov.br/DATASUS/index.php?area=0901&item=1&acao=25;^(12)^Instituto Brasileiro de Geografia e Estatística (IBGE). Diretoria de Pesquisas. Coordenação de Trabalho e Rendimento. Pesquisa nacional por amostra de domicílios: síntese de indicadores 2015. Rio de Janeiro: IBGE; 2016 [citado 2020 Abr 27]. Disponível em: https://biblioteca.ibge.gov.br/visualizacao/livros/liv98887.pdf;^(13)^Brasil. Ministério da Saúde. Departamento de Informática do Sistema Único de Saúde (DATASUS). População residente - Estudo de estimativas populacionais por município, idade e sexo 2000-2015 - Brasil. Brasília (DF): DATASUS; 2000 a 2013 [citado 2020 Abr 24]. Disponível em: http://tabnet.datasus.gov.br/cgi/tabcgi.exe?novapop/cnv/popbr;^(14)^Brasil. Ministério da Saúde. Departamento de Informática do Sistema Único de Saúde (DATASUS). Nascidos vivos - Brasil. Brasília (DF): DATASUS [citado 2020 Abr 24]. Disponível em: http://tabnet.datasus.gov.br/cgi/deftohtm.exe?sinasc/cnv/nvuf.def;^(15)^Departamento de Medicina Preventiva. Faculdade de Medicina da Universidade de São Paulo (FMUSP). Conselho Regional de Medicina da Estado de São Paulo (CREMESP). Conselho Federal de Medicina (CFM). Demografia médica no Brasil 2015. São Paulo: FMUSP, CREMESP, CFM; 2015 [citado 2020 Abr 24]. Disponível em: http://www.usp.br/agen/wp-content/uploads/DemografiaMedica30nov20153.pdf;^(16)^ Sociedade Brasileira de Quadril (SBQ). Busca de especialistas. São Paulo: SBQ; 2021 [citado 2020 Fev 24]. Disponível em: https://www.sbquadril.org.br/paciente/buscarespecialista/;^(17)^Sociedade Brasileira de Ortopedia Pediátrica (SBOP). Institucional/Busca de Especialistas. São Paulo: SBOP; 2021 [citado 2020 Abr 27]. Disponível em: https://www.sbop.org.br/encontre-um-especialista^(18)^

* Em 2015;

† Correlação de Pearson;

‡ significância bicaudal.

UF: unidade federativa; CNES: Cadastro Nacional de Estabelecimentos de Saúde; PNAD: Pesquisa Nacional por Amostra de Domicílios; IDH: Índice de Desenvolvimento Humano; IBGE: Instituto Brasileiro de Geografia e Estatística.

A taxa de tratamentos por mil nascidos vivos (por unidade federativa de residência) é mostrada na figura 3.Os círculos proporcionais indicam frequência absoluta de tratamentos, segundo a residência do paciente. A maior taxa foi encontrada no estado de Santa Catarina com 0,914 caso por mil nascidos vivos, e a menor em Amapá com 0,037 caso por mil nascidos vivos.

**Figura 3:** Taxa de casos por mil nascidos vivos e frequências absolutas por unidade federativa de residência no Grupo C. As cores do mapa representam as taxas de cirurgias para displasia do desenvolvimento do quadril no Grupo C por mil nascidos vivos por unidade federativa de residência dos pacientes conforme legenda

A figura 4 mostra a distribuição geográfica dos casos tratados no Grupo C em números absolutos (por unidade federativa de realização de cirurgia) e o fluxo de pacientes entre os estados e regiões. É possível observar que a Região Sudeste absorveu o maior número de pacientes de outras regiões, e que, proporcionalmente, os estados do Rio de Janeiro e do Maranhão, além do Distrito Federal, foram os que mais trataram pacientes de outros estados. Também foram identificados vários fluxos que atravessaram os limites das grandes regiões geográficas. Ainda, no período, foram registrados 333 fluxos de casos entre estados, sendo os maiores entre Minas Gerais e São Paulo (85 casos) e entre Goiás e o Distrito Federal (69 casos).

**Figura 4:** Fluxo de casos no Grupo C, segundo a unidade federativa de residência e a que realizou o tratamento. Para simplificação da imagem, apenas os fluxos dominantes são mostrados

## DISCUSSÃO

Em alguns países, a prevalência de tratamento cirúrgico da DDQ foi relatada como variando de 0,7 a 1,3 por mil nascidos vivos.^(19)^ Esse valor parece ser influenciado pela estratégia de triagem para identificação de casos precoces (com ou sem ultrassonografias), por fatores hereditários e raciais.^(19,20)^ Um estudo que avaliou a prevalência de intervenções cirúrgicas na DDQ (acetabuloplastia, osteotomia, osteotomia tripla, osteotomia periacetabular) em pacientes nascidos na Áustria, Alemanha e República Tcheca no período de 1992 a 2008 relatou redução de 46% no período de 16 anos.^(19)^ Naquele estudo, o uso rotineiro do exame ultrassonográfico de triagem foi implicado na diminuição na necessidade desse tipo de abordagem cirúrgica.^(19)^ Foi encontrada taxa de 0,29 caso de DDQ operado por mil nascidos vivos em nosso país (variando de 0,72 a 0,05, conforme a região), o que é inferior ao relatado na literatura.^(19)^ Essa frequência inferior de tratamentos merece ser discutida.

Primeiramente, este estudo não incluiu cirurgias realizadas na rede privada, o que pode subestimar a real frequência de tratamentos feitos no Brasil, onde cerca de 25,6% da população (dados de 2015) tinha acesso a planos de saúde. A distribuição geográfica heterogênea observada neste estudo confirma a hipótese inicialmente postulada por Puech,^(11)^ de que fatores raciais e demográficos impactam na epidemiologia da doença. A Região Sul do país, que possui grande proporção de migrantes europeus, teve taxa de tratamento para DDQ maior que o dobro da média nacional e 15 vezes maior que a da Região Norte. De fato, estudo genético em âmbito nacional encontrou prevalências de até 85% de ascendência europeia, em alguns municípios daquela região.^(21)^ A forte correlação entre a incidência de cirurgias e a proporção de indivíduos de raça branca nos estados, verificada neste estudo, reforça a impressão de que parte dessa correlação esteja relacionada a fatores hereditários.

No entanto, outros fatores parecem influenciar nessa distribuição. Como o estudo não mediu diretamente a prevalência da doença, mas sim a do tratamento de uma complicação da doença, os achados podem estar relacionados a iniquidades no acesso ao diagnóstico e ao tratamento. Verifica-se que regiões com menor aparato de saúde (menor taxa de especialistas) e piores índices socioeconômicos (renda *per capita* e IDH) possuem menores taxas de casos. Além disso, os estados do Sul e Sudeste receberam o maior influxo de pacientes, o que aumentou a polarização da realização de casos nesses centros. De fato, esse achado reflete uma violação aos princípios do SUS, cuja organização hierarquizada pressupõe que esse tipo de tratamento, na maioria dos casos, poderia ser oferecido em nível estadual ou, no máximo, regional. A lógica de tratamento fora do domicílio sobrecarrega os grandes centros, gera custos indiretos, atrapalha o desenvolvimento regional, desestimula a fixação de profissionais especializados no interior do país e fere o princípio de descentralização do SUS. Além disso, gera sofrimento às famílias que precisam se deslocar, em alguns casos, milhares de quilômetros com suas pequenas crianças para receber o tratamento adequado.^(22)^

A literatura indica a prevalência quatro a oito vezes maior de DDQ em mulheres.^(3,5,23)^ Neste estudo, o sexo feminino correspondeu a 67,8% dos casos, confirmando essa predominância no Brasil, em contraste com uma série de casos publicadas anteriormente em nosso meio, que relata maior prevalência do sexo masculino.^(10)^

Ao se analisarem os resultados obtidos em relação à distribuição cirúrgica racial da cirurgia da DDQ, observa-se uma clara predominância na raça branca (42,06%). Isso foi relatado em outros estudos em nosso país, embora em proporções diferentes. Em um estudo nacional, encontrou-se predominância na raça branca, com frequência de 81% dos casos.^(10)^ Essa diferença pode estar relacionada ao preenchimento incompleto das AIH no Brasil, pois em 41,6% dos casos incluídos neste estudo não existiam informações quanto à raça do paciente.

O tratamento cirúrgico da DDQ é mais prevalente entre crianças de 1 a 4 anos em nosso meio. Isso é compatível com a faixa etária mais comum para a indicação de tratamento cirúrgico da DDQ negligenciada.^(9)^Considerando os relatos da literatura de que, para cada 100 casos de DDQ, 19 evoluem para cirurgia,^(19)^ é possível estimar uma incidência Brasileira de DDQ de 1,52 por mil nascidos vivos (variando de 3,78 a 0,26 por mil nascidos vivos por região, sendo a maior em Santa Catarina). Isso quer dizer que a incidência estimada de casos de DDQ no Brasil é menor do que a de países mediterrâneos e do Leste Europeu, Japão, Austrália e Nova Zelândia, e maior que alguns países africanos e do Oeste Europeu, além de manter semelhança com alguns estados dos Estados Unidos e outros países americanos, como o Chile.^(24)^

As diferenças encontradas nas frequências de tratamentos em diferentes grupos (estratégias de busca), embora revelem distintas especificidades de cada parâmetro de busca, também levam a discutir a importância do correto preenchimento das AIH. Como as informações deste estudo derivam de dados secundários, fatores que influenciam no preenchimento e no registro das AIH, podem interferir nos resultados. Entre esses fatores, são citados na literatura a completude e correção do preenchimento pelo médico; a dificuldade em decifrar a letra do médico; e a falta de treinamento e o desconhecimento, por parte dos funcionários, dos hospitais das regras de codificação.^(25)^ Exemplo dessa situação foi observado neste trabalho, em que a raça dos pacientes não foi documentada em mais de 40% dos casos.

Há limitações neste estudo. Por se tratar de estudo ecológico, não existe a pretensão de se mostrarem relações causais, mas simplesmente analisar fatores de associação. No entanto, por explorar uma base de dados robusta de amplitude nacional, este estudo traz à tona informações inéditas a respeito da epidemiologia brasileira da DDQ e suas complicações, cujo impacto individual na saúde de seus portadores e na de suas famílias pode ser devastador. Um potencial viés, é relacionado à qualidade dos dados originais das AIH. O uso de três estratégias diferentes de pesquisa (Grupos A, B e C) permitiu selecionar dados mais específicos ao objeto da pesquisa. No entanto, não é possível identificar todos os casos de erro de preenchimento, omissões ou fraudes. Além das informações originais contidas neste estudo, ele pode ajudar a conscientizar os médicos da importância de alimentar as bases de dados oficiais, preenchendo corretamente as AIH, e, consequentemente, fornecendo informações epidemiológicas confiáveis.

## CONCLUSÃO

O tratamento cirúrgico da displasia do desenvolvimento do quadril no Brasil é frequente, gera custos relevantes e é distribuído de maneira heterogênea e desigual no Sistema Único de Saúde. Os estados do Sul têm as maiores incidências de casos. Fatores raciais e socioeconômicos estão associados a essa distribuição. Houve pequena variação temporal na incidência de casos operados no período de uma década.
